# Evidence for *PTGER4*,*PSCA,* and *MBOAT7* as risk genes for gastric cancer on the genome and transcriptome level

**DOI:** 10.1002/cam4.1719

**Published:** 2018-09-06

**Authors:** Sophie K. M. Heinrichs, Timo Hess, Jessica Becker, Lutz Hamann, Yogesh K. Vashist, Katja Butterbach, Thomas Schmidt, Hakan Alakus, Iurii Krasniuk, Aksana Höblinger, Philipp Lingohr, Monika Ludwig, Alexander F. Hagel, Claus W. Schildberg, Lothar Veits, Ugne Gyvyte, Katharina Weise, Vitalia Schüller, Anne C. Böhmer, Julia Schröder, Jan Gehlen, Nicole Kreuser, Sebastian Hofer, Hauke Lang, Florian Lordick, Peter Malfertheiner, Markus Moehler, Oliver Pech, Nikolaos Vassos, Ernst Rodermann, Jakob R. Izbicki, Martin Kruschewski, Katja Ott, Ralf R. Schumann, Michael Vieth, Elisabeth Mangold, Evita Gasenko, Limas Kupcinskas, Hermann Brenner, Peter Grimminger, Luis Bujanda, Federico Sopeña, Jesús Espinel, Concha Thomson, Ángeles Pérez‐Aísa, Rafael Campo, Fernando Geijo, Daniela Collette, Christiane Bruns, Katharina Messerle, Ines Gockel, Markus M. Nöthen, Hans Lippert, Karsten Ridwelski, Angel Lanas, Gisela Keller, Michael Knapp, Marcis Leja, Juozas Kupcinskas, Maria A. García‐González, Marino Venerito, Johannes Schumacher

**Affiliations:** ^1^ Institute of Human Genetics University of Bonn Bonn Germany; ^2^ Department of Genomics, Life & Brain Center University of Bonn Bonn Germany; ^3^ Institute for Microbiology and Hygiene Charité University Medical Center Berlin Berlin Germany; ^4^ Department of General, Visceral and Thoracic Surgery University Medical Center Hamburg‐Eppendorf Hamburg Germany; ^5^ Division of Clinical Epidemiology and Aging Research German Cancer Research Center (DKFZ) Heidelberg Germany; ^6^ Department of General, Visceral and Transplantation Surgery University of Heidelberg Heidelberg Germany; ^7^ Department of General, Visceral and Cancer Surgery University of Cologne Cologne Germany; ^8^ Department of General and Visceral Surgery Municipal Hospital Solingen Solingen Germany; ^9^ Department of Internal Medicine I Community Hospital Mittelrhein Koblenz Germany; ^10^ Department of General, Visceral, Thoracic and Vascular Surgery University of Bonn Bonn Germany; ^11^ Association for Oncological Studies (Gefos) Dortmund Germany; ^12^ Department of Medicine I, Gastroenterology and Interventional Endoscopy University of Erlangen Erlangen Germany; ^13^ Department of Surgery University of Erlangen Erlangen Germany; ^14^ Institute of Pathology Klinikum Bayreuth Bayreuth Germany; ^15^ Department of Gastroenterology and Institute for Digestive Research Lithuanian University of Health Sciences Kaunas Lithuania; ^16^ Institute for Medical Biometry, Informatics and Epidemiology University of Bonn Bonn Germany; ^17^ Department of Visceral, Transplant, Thoracic and Vascular Surgery University Hospital of Leipzig Leipzig Germany; ^18^ Department of General, Visceral and Transplant Surgery University Medical Center University of Mainz Mainz Germany; ^19^ University Cancer Center Leipzig (UCCL) University Hospital of Leipzig Leipzig Germany; ^20^ Department of Gastroenterology, Hepatology and Infectious Diseases Otto‐von‐Guericke University Hospital Magdeburg Germany; ^21^ First Medical Clinic and Policlinic University Medical Center University of Mainz Mainz Germany; ^22^ Department of Gastroenterology and Interventional Endoscopy St. John of God Hospital Regensburg Germany; ^23^ Association of Medical Practices in Hematology and Internal Oncology Troisdorf Germany; ^24^ Department of General and Visceral Surgery Hospital Frankfurt Oder Frankfurt Oder Germany; ^25^ Department of General, Visceral and Thorax Surgery RoMed Hospital Rosenheim Rosenheim Germany; ^26^ Institute of Clinical and Preventive Medicine University of Latvia Riga Latvia; ^27^ Riga East University Hospital Riga Latvia; ^28^ Division of Preventive Oncology German Cancer Research Center (DKFZ) and National Center for Tumor Diseases (NCT) Heidelberg Germany; ^29^ German Cancer Consortium (DKTK) German Cancer Research Center (DKFZ) Heidelberg Germany; ^30^ CIBER de Enfermedades Hepáticas y Digestivas (CIBERehd) Madrid Spain; ^31^ Department of Gastroenterology Hospital Donostia/Instituto Biodonostia Universidad del País Vasco (UPV/EHU) San Sebastián Spain; ^32^ Instituto de Investigación Sanitaria Aragón (IIS Aragón) Zaragoza Spain; ^33^ Department of Gastroenterology Hospital Clínico Universitario Lozano Blesa Zaragoza Spain; ^34^ Department of Gastroenterology Complejo Hospitalario León Spain; ^35^ Department of Gastroenterology Hospital Obispo Polanco Teruel Spain; ^36^ Department of Gastroenterology Hospital del Sol Marbella Spain; ^37^ Department of Gastroenterology Hospital Parc Tauli Sabadell Spain; ^38^ Department of Gastroenterology Hospital Clínico Universitario Salamanca Spain; ^39^ Association of Medical Practices in Hematology and Oncology Dortmund Germany; ^40^ An‐Institute for Quality Control in Surgery Otto‐von‐Guericke University Hospital Magdeburg Germany; ^41^ Department of Surgery Hospital Magdeburg Magdeburg Germany; ^42^ Institute of Pathology Technical University of Munich Munich Germany; ^43^ Instituto Aragonés de Ciencias de la Salud (IACS) Zaragoza Spain; ^44^ Center of Human Genetics, University Hospital Marburg Marburg Germany

**Keywords:** eQTL study, gene expression, genetic association study, stomach neoplasms

## Abstract

Genetic associations between variants on chromosome 5p13 and 8q24 and gastric cancer (GC) have been previously reported in the Asian population. We aimed to replicate these findings and to characterize the associations at the genome and transcriptome level. We performed a fine‐mapping association study in 1926 GC patients and 2012 controls of European descent using high dense SNP marker sets on both chromosomal regions. Next, we performed expression quantitative trait locus (eQTL) analyses using gastric transcriptome data from 143 individuals focusing on the GC associated variants. On chromosome 5p13 the strongest association was observed at rs6872282 (*P* = 2.53 × 10^−04^) and on chromosome 8q24 at rs2585176 (*P* = 1.09 × 10^−09^). On chromosome 5p13 we found cis‐eQTL effects with an upregulation of PTGER4 expression in GC risk allele carrier (*P* = 9.27 × 10^−11^). On chromosome 8q24 we observed cis‐eQTL effects with an upregulation of PSCA expression in GC risk allele carrier (*P* = 2.17 × 10^−47^). In addition, we found trans‐eQTL effects for the same variants on 8q24 with a downregulation of MBOAT7 expression in GC risk allele carrier (*P* = 3.11 × 10^−09^). In summary, we confirmed and refined the previously reported GC associations at both chromosomal regions. Our data point to shared etiological factors between Asians and Europeans. Furthermore, our data imply an upregulated expression of PTGER4 and PSCA as well as a downregulated expression of MBOAT7 in gastric tissue as risk‐conferring GC pathomechanisms.

## 1 INTRODUCTION

The majority of gastric adenocarcinomas, here called gastric cancer (GC), is sporadic and has a multifactorial and heterogeneous etiology. According to its localization, GC is subdivided into a cardia and noncardia type and according to the histopathological classification of Lauren, GC is subdivided into a diffuse, intestinal or mixed type.^1^ On the etiological level, inflammation represents an important risk factor, which is mainly caused by *Helicobacter pylori* (*H. pylori*) infection.^1^

In multifactorial diseases, genome‐wide association studies (GWAS) have systematically led to the identification of risk loci involved in disease etiology. Accordingly, GWAS in GC have been carried out in the Asian population, which have led to the identification of risk loci on chromosome 1q22, 3q13, 5p13, 6p21, 7p15, 8q24, and 10q23.^2^, ^3^, ^4^, ^5^, ^6^ The risk loci on 5p13^7^ and 8q24^8^, ^9^ have been confirmed to contribute to GC development also in the European population. However, functional data on the underlying risk genes and on disease conferring pathomechanisms at these loci are scarce. Thus, we aimed to further confirm the role of 5p13 and 8q24 in the etiology of GC in Europeans and to characterize the associations on the genome and transcriptome level. However, it should be noted, that the locus on chromosome 1q22 was also independently replicated in an European GC sample under certain genetic models (dominant and recessive).^10^ Because no allelic association tests were presented in this study we did not follow up the association at the 1q22 locus.

## 2 MATERIALS AND METHODS

### 2.1 Samples

For the fine‐mapping association study at 5p13 and 8q24, we used a sample consisting of 1926 histopathologically confirmed GC patients (680 females, 1246 males) and 2012 ethnically matched controls (985 females, 1027 males) that were not screened for the presence of GC. All study subjects were of European descent and were recruited at four different study sites in Latvia, Lithuania, Spain and Germany. Table S1 provides sample details, including origin of samples and distribution of GC localization (cardia, noncardia) as well as Lauren type (diffuse, intestinal, and mixed). Informed consent was obtained from all participants and approval was obtained from the ethic board of each participating site.

The expression quantitative trait locus (eQTL) analysis was based on 143 individuals of German descent (15 females, 128 males). All subjects were histopathologically diagnosed with intestinal metaplasia of the distal esophagus (Barrett's esophagous) and additionally, endoscopic biopsies of the gastric cardia were obtained during routine surveillance gastroscopies. Absence of pathological changes and *H. pylori* infection in the gastric mucosa was histopathologically confirmed for all cases. Informed consent was obtained from all participants as well as approval from the responsible ethic board.

### 2.2 Genotyping and expression analyses

For the fine‐mapping association study on 5p13 and 8q24, single‐nucleotide polymorphisms (SNPs) were selected for genotyping with the aim to impute a maximum number of genetic variants within both regions. To define the regions of interest the main recombination hotspots flanking both loci were determined using Haploview Version 4.2 and the HapMap dataset III (Release August 2010).^11^ Next, all SNPs with a minor allele frequency (MAF) of ≥5% on Illumina Human Core Exome Chip were used to reduce the number of necessary tagging SNPs within both regions. To prove that these SNPs cover the regions sufficiently an imputation on a genome‐wide genotyped test data set^12^ was performed. Finally, we used the test data set and ensured that all common publicly released SNPs within both regions were imputed with high imputation quality scores (INFO > 0.4). This resulted in 14 SNPs located within 5p13 and 6 SNPs within 8q24 (Table 1) as tagging markers for the respective loci. All 20 SNP markers were genotyped using a Sequenom MassARRAY iPlex Gold^®^ system (Sequenom, San Diego, CA, USA). For quality control (QC), intra‐ and interplate duplicates were included. Furthermore, negative controls (H2O) were added to each 384 well plate in order to exclude contamination. Cluster plots of all SNPs were visually checked and manually corrected if necessary. The postgenotype QC comprised the exclusion of SNPs with Hardy‐Weinberg equilibrium (HWE) *P* < 1 × 10^−04^ in controls and *P* < 1 × 10^−06^ in patients, a call rate (CR) < 95% as well as exclusion of samples with a CR < 90%. A single marker (rs138377917 on chromosome 8q24) failed QC. Detailed information on primer sequences, genotyping and genotype calling is available upon request.

**Table 1:** Case‐control comparison (1926 GC cases, 2012 controls) of genotyped SNPs at chromosomal regions 5p13 and 8q24. One marker (rs138377917 on chromosome 8q24) failed QC and is not shown

SNP	Chr	Allele^a^	RR (95% CI)	Combined *P*‐value
rs7716982	5	G/T	1.06 (0.96‐1.16)	2.31 × 10^−01^
rs6893430	5	C/T	1.15 (1.03‐1.29)	1.20 × 10^−02^
rs6861121	5	A/G	1.07 (0.94‐1.23)	3.07 × 10^−01^
rs7726237	5	G/A	1.12 (1.02‐1.23)	1.89 × 10^−02^
rs10737963	5	C/A	1.08 (0.97‐1.19)	1.60 × 10^−01^
rs12523329	5	C/T	1.06 (0.96‐1.17)	2.79 × 10^−01^
rs1002424	5	A/G	1.16 (1.05‐1.29)	3.09 × 10^−03^
rs257009	5	C/T	1.09 (0.99‐1.20)	8.39 × 10^−02^
rs10053664	5	C/T	1.06 (0.97‐1.16)	2.23 × 10^−01^
rs13361707	5	C/T	1.17 (1.06‐1.29)	2.29 × 10^−03^
rs3805486	5	A/G	1.13 (0.99‐1.29)	6.20 × 10^−02^
rs462366	5	T/C	1.04 (0.94‐1.15)	4.28 × 10^−01^
rs6876367	5	C/T	1.12 (1.01‐1.24)	2.70 × 10^−02^
rs2291782	5	A/G	1.06 (0.96‐1.16)	2.57 × 10^−01^
rs2976400	8	A/G	1.04 (0.95‐1.15)	3.70 × 10^−01^
rs2976392	8	A/G	1.29 (1.18‐1.41)	2.14 × 10^−08^
rs2976397	8	T/G	1.30 (1.19‐1.42)	7.18 × 10^−09^
rs12155758	8	A/G	1.24 (1.13‐1.37)	1.03 × 10^−05^
rs1435453	8	C/T	1.23 (1.12‐1.34)	6.98 × 10^−06^

Chr, chromosome; CI, confidence interval; RR, relative risk.

a The underlined allele represents the GC risk allele.

For the eQTL analysis DNA from 143 donors was extracted from peripheral blood and genotyped genome‐wide using Human OmniExpress‐v1.1 and HumanOmniExpressExome‐v1.2 BeadChips (Illumina, San Diego, CA, USA). The postgenotype QC comprised the exclusion of SNPs with HWE *P* < 1 × 10^−05^, minor allele frequency (MAF) <5% or a CR < 98% as well as the exclusion of samples with a CR < 99%. For the expression analysis, total RNA from gastric cardia biopsies was isolated using the AllPrep DNA/RNA Mini Kit (Qiagen, Hilden, Germany). The transcriptome was assessed using the HumanHT‐12v4 Expression BeadChip (Illumina) that targets more than 48 000 transcripts in the refseq database (Build 36.2, release 22). Only probes with a *P*_detection_ < 0.01 in more than 5% of the samples were included for analysis. Furthermore, all probes were filtered for unique alignment and quality. Probes classified with a perfect or good quality as reported in the R package illuminaHumanv4.db were considered for further analysis.

### 2.3 Fine‐mapping and eQTL analyses

For the fine‐mapping association analysis, all genotyped SNPs were used for the imputation of additional markers at both regions. For this purpose, IMPUTE2^13^ was used utilizing the 1000 Genomes Phase 3 data as reference.^14^ After imputation, all SNPs with an info score >0.7 were further processed, which resulted in 478 SNPs on 5p13 and 315 SNPs on 8q24. For each of the four study sites, association analysis of genotyped and imputed SNPs was performed by SNPTEST v2.5.^15^ A fixed‐effects meta‐analysis was performed to combine the results across study sites. We additionally performed genotype‐phenotype (GxP) analyses and stratified our cases according to tumor localization (cardia, noncardia) as well as histopathological Lauren type (diffuse, intestinal, or mixed). Moreover, pairwise linkage disequilibrium (LD) between markers was determined using SNAP^16^ and the 1000 Genomes Pilot 1 data from the European population.^14^

For the eQTL analysis genotypes of 568 265 autosomal SNPs and expression intensity data from 11 900 probes were used from all 143 probands. The expression data were quantile normalized and eQTLs were mapped using a linear regression model implemented in the MatrixEQTL R package.^17^ The associations were corrected for the top five principal components and eQTL effects with a false discovery rate (FDR) of <0.05 were considered as significant. In addition, eQTLs with a distance of <1 Mb to the corresponding probes were considered as cis‐regulatory variants. Vice versa, SNPs with a distance of >1 Mb to the corresponding probes were classified as trans‐eQTLs.

## 3 RESULTS

### 3.1 Association and eQTL findings on chromosome 5p13

**Figure 1:**
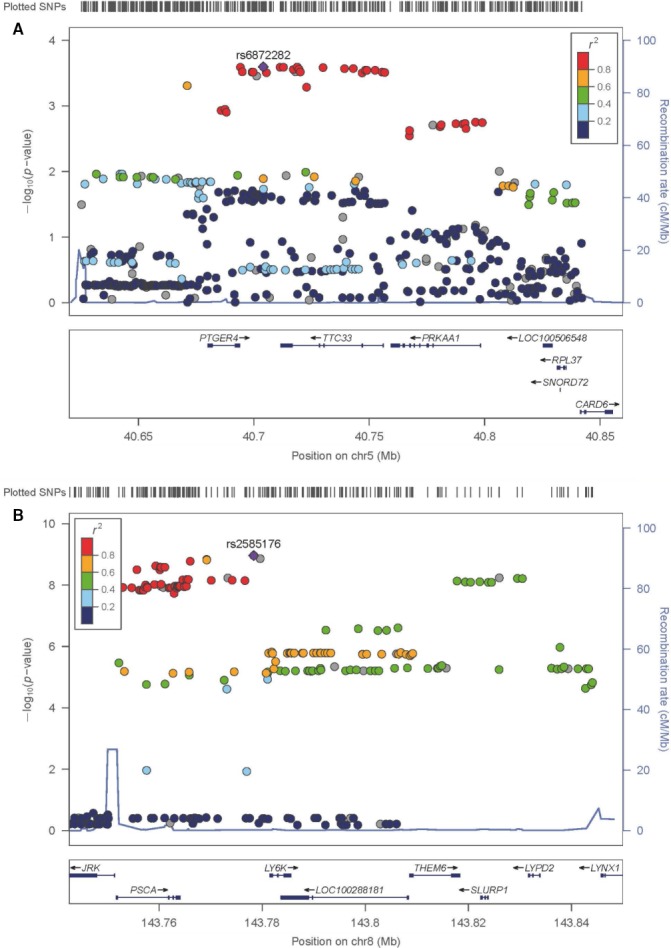
Regional association plots of GC associations at chromosome 5p13 (A) and chromosome 8q24 (B). SNP association results are shown as ‐log P. The most significant associated SNP—rs6872282 at 5p13 (A), rs2585176 at 8q24 (B)—is shown as solid diamond. Pair‐wise correlation (*r* ^2^) between the most significant associated SNP and the other SNPs in a 500 kb flanking region is illustrated by the color scheme. The blue spikes show the estimated recombination rates. All annotated genes in both regions are shown at the bottom and their reading direction is given by arrows

Of all genotyped SNPs at 5p13 5 variants showed significant GC association (Table 1). Table S2 shows the association findings within each study site. The strongest association in the entire sample was observed for rs13361707 (*P* = 2.29 × 10^−03^, RR = 1.17 (95% CI = 1.06‐1.29)). The fine‐mapping at this region included 478 imputed SNPs, of which 28 showed GC association. The strongest association was observed for rs6872282 (*P* = 2.53 × 10^−04^, RR = 1.22 (95% CI = 1.09‐1.35), risk allele C, opposite allele T, see Table S3). Of note, rs13361707 and rs6872282 are in high LD with *r* ^2^ = 0.92. Figure 1A provides the regional association plot at 5p13 and shows that all associated variants encompass the coding regions of the genes PTGER4, TTC33, and PRKAA1. The GxP analysis at 5p13 using GC tumor localization and histopathological Lauren type as strata did not lead to an association improvement. Table S4 shows all GxP results for rs6872282, the strongest associated marker in the entire analysis. In addition, a conditional analysis using rs6872282 did not reveal any independent disease association (*P* > 0.5, data not shown).

**Figure 2:**
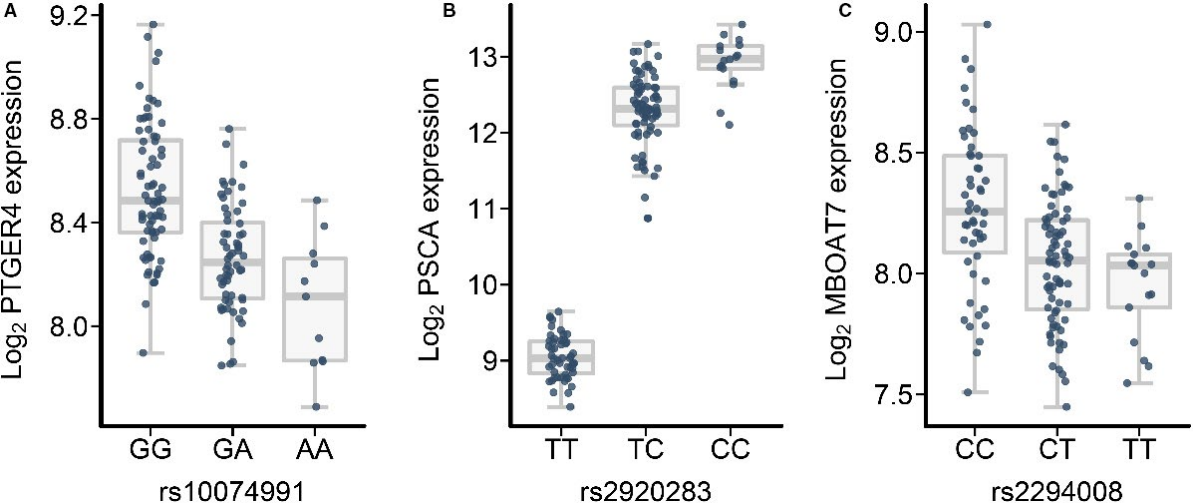
eQTL effects for GC associated variants at chromosomal regions 5p13 and 8q24. Log2 gene expression, error bars for median log2 expression and standard deviation are shown as box plot (*y* axis) sorted by SNP genotype (*x*‐axis) with the common allele on the left. The individual log2 gene expression is indicated by small dots in blue (*y*‐axis). A, cis‐eQTL (rs10074991, risk allele G) for the expression of PTGER4 at 5p13 (*P* = 9.27 × 10^−11^, regression slope (*β*) = −56.18). B, cis‐eQTL (rs2920283, risk allele C) for the expression of PSCA (*P* = 2.17 × 10^−47^, *β* = 3958.10). C, trans‐eQTL (rs2294008, risk allele T) for the expression of MBOAT7 (*P* = 1.99 × 10^−09^, *β* = −40.57)

The eQTL analysis at 5p13 was initially restricted to SNPs with *r* ^2^ > 0.8 to the most associated GC variant and revealed cis‐eQTL effects for the expression of PTGER4 in gastric tissue (*P* = 9.27 × 10^−11^ for rs10074991). Here, an upregulated expression of the transcript was observed in GC risk allele carrier (Figure 2A). We then tested all 5p13 SNPs for PTGER4 eQTL effects, which revealed that rs10074991 represents the strongest eQTL for the expression of PTGER4 at this locus. Finally, we did not observe any trans‐eQTL effects using GC associated risk variants at 5p13 and an FDR < 0.05.

### 3.2 Association and eQTL findings on chromosome 8q24

Of all genotyped SNPs at 8q24 4 variants showed significant GC association (Table 1). Table S2 shows the association findings within each study site. The strongest association in the entire sample was observed for rs2976397 (*P* = 7.18 × 10^−09^, RR = 1.30 (95% CI = 1.19‐1.42)). The fine‐mapping at 8q24 included 315 imputed SNPs. Of them 42 SNPs showed GC association with rs2585176 being the most associated variant (*P* = 1.09 × 10^−09^, RR = 1.34 (95% CI = 1.22‐1.47), risk allele T, opposite allele A, see Table S3). Notably, rs2976397 and rs2585176 are in high LD with *r* ^2^ = 0.90. Figure 1B shows the regional association plot at 8q24 and shows that all associated variants are located close to the genes JRK, PSCA, and LY6K. The GxP analysis at 8q24 using GC tumor localization and histopathological Lauren type as strata did not lead to the identification of a particular GC subtype with predominant association. Table S5 shows all GxP results for rs2585176, the strongest associated marker in the entire analysis. In addition, a conditional analysis using rs2585176 did not reveal any independent disease association (*P* > 0.5, data not shown).

The eQTL analysis at 8q24 was initially restricted to SNPs with *r* ^2^ > 0.8 to the most associated GC variant and revealed cis‐eQTL effects for the expression of PSCA (*P* = 2.17 × 10^−47^). The lead eQTL is rs2920283 and an upregulated PSCA expression in gastric tissue was observed in GC risk allele carrier (Figure 2B). As for 5p13, we then tested all 8q24 SNPs for PSCA eQTL effects, which revealed that rs2920283 represents the strongest eQTL for the expression of PSCA at this locus. Finally, we tested for trans‐eQTL effects using GC associated risk variants at 8q24 with *r* ^2^ > 0.8 to the lead GC SNP. Here, we observed a regulatory effect for the expression of MBOAT7 in gastric tissue, which is located on chromosome 19q13. The most significant trans‐eQTL was rs2294008 (*P* = 1.99 × 10^−09^) and led to a downregulated expression of MBOAT7 in GC risk allele carrier [Figure 2C]. Notably, the lead cis‐ and trans‐eQTLs are in perfect LD (*r* ^2^ = 1.00) pointing to a common regulatory effect. However, it should be noted that the statistical significance of the cis‐eQTL is much higher than for the trans‐eQTL.

## 4 DISCUSSION

The GC associations at 5p13 and 8q24 have been initially reported in the Asian population.^2^, ^4^ It has subsequently been shown that both loci also play a role in GC etiology in European populations.^7^, ^8^, ^9^ In the present study, we aimed to further replicate the GC association in the largest so far analyzed European cohort as well as to refine and characterize the association signals on the genome and transcriptome level.

On chromosome 5p13 all GC associated variants were in high LD (*r* ^2^ > 0.8) to the most associated marker (rs6872282) and in high LD (*r* ^2^ > 0.8) to all risk SNPs previously reported at this locus in Asians^2^, ^18^, ^19^, ^20^, ^21^, ^22^, ^23^ and Europeans.^7^ Thus, together with previous findings, our data provide evidence that one or more of these SNPs represent(s) the true GC conferring variant(s) in both the Asian and European population. Of the implicated SNPs rs3805495 is in perfect LD to the leading GC risk variant rs6872282 (*r* ^2^ = 1) and represents an interesting functional SNP. According to RegulomeDB^24^ this variant is predicted to alter motifs for several transcription factors and to affect histone modification in digestive tissues including gastric mucosa. In addition, we found that the GC risk alleles of the associated SNPs lead to an upregulated PTGER4 expression in gastric tissue, which represents a plausible GC pathomechanism at this locus. To our knowledge, this eQTL effect has not been reported so far and thus needs further replication in independent gastric tissue samples.

Also on the functional level, PTGER4 represents an interesting candidate for GC. The gene encodes the prostaglandin E2 (PGE2) receptor 4 (EP4), which mediates cellular responses to PGE2. PGE2 is derived from arachidonic acids through the enzymatic activity of cyclooxygenase‐2 (COX‐2). It has already been shown that the COX‐2/PGE2 pathway plays a key role in generation of the inflammatory microenvironment in GC and represents a downstream effector of Toll‐like receptor (TLR) activation that is—among others—triggered through *H. pylori* infection,^25^ the most prominent environmental GC risk factor. Interestingly, one study with *H. pylori* positive and negative GC cases and controls found a gene‐environment interaction between 5p13 risk SNPs and *H. pylori* infection that showed association with GC.^18^

Genetic variability at 5p13 also contributes to other diseases with inflammatory components, including Crohn's disease,^26^, ^27^, ^28^, ^29^, ^30^, ^31^ ulcerative colitis,^32^, ^33^, ^34^ ankylosing spondylitis^35^ and multiple sclerosis.^36^, ^37^, ^38^ Furthermore, cis‐regulatory effects involving the expression of PTGER4 in some of these traits have been described previously in lymphoblastoid cell lines.^39^ However, all SNPs and eQTLs implicated in these studies are only in weak LD (*r* ^2^ < 0.3) to the GC variants and PTGER4‐eQTLs identified in the present study (see Table S6). The findings point to PTGER4 as risk gene for different diseases with inflammatory components. However, more complex pathomechanisms with disease‐ and/or tissue‐specific effects on the PTGER4 expression regulation seem to be present at this locus.

On chromosome 8q24 all GC associated variants were in high LD (*r* ^2^ > 0.8) to the most associated marker (rs2585176) and in high LD (*r* ^2^ > 0.8) to all risk SNPs previously reported at this locus in Asians and Europeans (summarized in Ref. 40). As for 5p13, this provides evidence that shared etiological factors between Asians and Europeans contribute to GC risk at this locus and that one or more of the identified SNPs represent(s) the true GC conferring variant(s). Of the implicated SNPs rs2294008 (C>T, Met1Thr) represents an interesting functional variant, which is located in the translation starting site of PSCA, is in high LD (*r* ^2^ = 0.86) with the lead GC SNP and also the strongest trans‐eQTL for the expression of MBOAT7 (see below). This SNP leads to an alternative PSCA splice form on the protein level^41^ and thus has been favored as true GC risk variant at this locus. In addition, rs2585183 represents an interesting functional variant according to RegulomeDB.^24^ The variant is in high LD to the leading GC risk SNP (*r* ^2^ = 0.90) and is predicted to affect histone modification in digestive tissues including gastric mucosa.

Moreover, we found that the risk alleles of the GC associated markers lead to an upregulated expression of PSCA. The presence of this eQTL in gastric tissue is supported by GTEx (version 7, dbGaP accession phs000424.v7.p2), where the strongest eQTL in our study (rs2920283) shows cis‐regulatory effects for PSCA with *P* = 8.1 × 10^−33^. Furthermore, cis‐eQTL effects involving the presented variants and PSCA have been shown previously in healthy gastric and gastric tumor tissue.^42^ Thus, the PSCA eQTL has been independently validated and represents a plausible GC pathomechanism at 8q24.

Also on the functional level PSCA represents an interesting candidate for GC development. The gene encodes a glycosylphosphatidylinositol‐anchored cell‐surface protein with highest expression in tumor cells and seems to play a role in multiple cellular processes, including immune‐modulation, cell adhesion, proliferation and survival.^43^ The PSCA risk alleles of the GC associated SNPs also mediate risk for chronic atrophic gastritis,^9^, ^44^ an *H. pylori* ‐induced precursor lesion of GC. In contrast, the opposite alleles at the same variants lead to duodenal ulcer,^41^, ^44^, ^45^, ^46^ which is also induced by *H. pylori* infection, but characterized by severe antral mucosa inflammation and protective GC effects.^47^, ^48^, ^49^ Thus, it has been hypothesized that gastric *H. pylori* infection leads to chronic atrophic gastritis and GC in patients with GC risk alleles at PSCA variants, whereas carrier of the opposite alleles tend to develop severe antral mucosa inflammation and duodenal ulcer.^41^, ^44^, ^45^, ^46^

The same regulatory variants on 8q24 that represent cis‐eQTLs for the expression of PSCA lead to a downstream downregulated MBOAT7 expression in gastric tissue, which suggests that MBOAT7 might be also of relevance in GC etiology. Interestingly, MBOAT7 encodes an enzyme with lysophosphatidylinositol acyltransferase activity and has been implicated in anti‐inflammatory processes through the regulation of arachidonic acid‐derived prostaglandin (PG) levels.^50^ Recently, it has been shown that MBOAT7 plays a pivotal role in hepatic inflammation and fibrosis in patients with hepatitis C infection^51^ and alcohol‐related liver cirrhosis.^52^ Thus, MBOAT7 might also contribute to GC susceptibility via biological pathways that are involved in inflammation. However, although the expression of PSCA and MBOAT7 is regulated by the same SNPs, a cellular connection between them has not been described so far.

In conclusion, our study has confirmed the role of 5p13 and 8q24 in the etiology of GC in Europeans and has characterized the associations on the genome and transcriptome level. Whereas the eQTL effect of PSCA has been described before, the eQTL effects involving PTGER4 and MBOAT7 have not been described before in the context of GC. Future work is now required as to whether the same eQTL effects are present in the remaining gastric regions or other tissues with relevance in GC development. Additionally, functional work is required to prove that the observed eQTL effects play a role in GC development rather than represent epiphenomena with no relevance for GC development. In this context, our fine‐mapping association and eQTL study with altered gastric expression of PTGER4, PSCA, and MBOAT7 in GC risk allele carriers may serve as impetus.

## CONFLICT OF INTEREST

None declared.
