# Supplementary material for: Evidence for PTGER4,PSCA, and MBOAT7 as risk genes for gastric cancer on the genome and transcriptome level
Source: Cancer Med. 2018 Sep 6;7(10):5057–65. doi: 10.1002/cam4.1719 (PMC6198243; doi:10.1002/cam4.1719)
Supplement: Supplementary file 1 [file CAM4-7-5057-s001.doc]

**Supplementary InfoRmation**

**Supplementary Table 1:** Sample overview of GC patients **(a)** and controls **(b)**.

**1a)** Patients

| **Origin** | **Male** | **Female** | **Total** | **Localization** | | | **Lauren-Type** | | | |
| --- | --- | --- | --- | --- | --- | --- | --- | --- | --- | --- |
|  |  |  |  | **non-cardia** | **cardia** | **unknown** | **diffuse** | **intestinal** | **mixed** | **unknown** |
| **Latvia** | 181 | 115 | 296 | - | - | 296 | 55 | 74 | 54 | 113 |
| **Lithuania** | 105 | 54 | 159 | 107 | 10 | 42 | 70 | 61 | 10 | 18 |
| **Spain** | 352 | 177 | 529 | 426 | 103 | - | 164 | 244 | 40 | 81 |
| **Germany** | 608 | 334 | 942 | 656 | 198 | 88 | 311 | 409 | 77 | 145 |
| **Total** | 1,246 | 680 | 1,926 | 1,189 | 311 | 426 | 600 | 788 | 181 | 357 |

**1b)** Controls

| **Origin** | **Male** | **Female** | **Total** |
| --- | --- | --- | --- |
|  |  |  |  |
| **Latvia** | 96 | 242 | 338 |
| **Lithuania** | 79 | 101 | 180 |
| **Spain** | 389 | 180 | 569 |
| **Germany** | 463 | 462 | 925 |
| **Total** | 1,027 | 985 | 2,012 |

**Supplementary Table 2:** Case control comparison (1,926 GC cases, 2,012 controls) of genotyped SNPs at chromosomal regions 5p13 and 8q24.

**2a)** Case control sample with Latvian origin (296 GC cases, 338 controls)

| **SNP** | **Chr** | **Allele^1^** | **MAF (%) cases** | **MAF (%) controls** | **p-value** | **RR (95% CI)** |
| --- | --- | --- | --- | --- | --- | --- |
| rs7716982 | 5 | G/T | 0.33 | 0.32 | 8.37 x 10^-01^ | 1.03 (0.81-1.30) |
| rs6893430 | 5 | T/C | 0.17 | 0.21 | 8.17 x 10^-02^ | 0.78 (0.58-1.03) |
| rs6861121 | 5 | G/A | 0.10 | 0.09 | 3.39 x 10^-01^ | 1.20 (0.82-1.76) |
| rs7726237 | 5 | G/A | 0.34 | 0.30 | 1.68 x 10^-01^ | 1.18 (0.93-1.50) |
| rs10737963 | 5 | C/A | 0.24 | 0.23 | 8.64 x 10^-01^ | 1.02 (0.79-1.33) |
| rs12523329 | 5 | C/T | 0.25 | 0.25 | 8.95 x 10^-01^ | 0.98 (0.76-1.27) |
| rs1002424 | 5 | G/A | 0.24 | 0.28 | 9.67 x 10^-02^ | 0.81 (0.63-1.04) |
| rs257009 | 5 | C/T | 0.30 | 0.28 | 3.39 x 10^-01^ | 1.12 (0.88-1.43) |
| rs10053664 | 5 | C/T | 0.46 | 0.44 | 5.18 x 10^-01^ | 1.08 (0.86-1.34) |
| rs13361707 | 5 | T/C | 0.24 | 0.28 | 8.89 x 10^-02^ | 0.80 (0.62-1.03) |
| rs3805486 | 5 | G/A | 0.11 | 0.11 | 9.43 x 10^-01^ | 0.99 (0.69-1.41) |
| rs462366 | 5 | T/C | 0.25 | 0.26 | 8.87 x 10^-01^ | 0.98 (0.76-1.27) |
| rs6876367 | 5 | T/C | 0.22 | 0.26 | 1.43 x 10^-01^ | 0.83 (0.64-1.07) |
| rs2291782 | 5 | A/G | 0.32 | 0.31 | 4.57 x 10^-01^ | 1.10 (0.86-1.39) |
| rs2976400 | 8 | A/G | 0.29 | 0.30 | 6.68 x 10^-01^ | 0.95 (0.74-1.21) |
| rs2976392 | 8 | G/A | 0.35 | 0.49 | 2.33 x 10^-07^ | 0.57 (0.45-0.71) |
| rs2976397 | 8 | G/T | 0.36 | 0.50 | 1.87 x 10^-07^ | 0.57 (0.45-0.71) |
| rs12155758 | 8 | A/G | 0.45 | 0.34 | 3.81 x 10^-05^ | 1.57 (1.25-1.97) |
| rs1435453 | 8 | T/C | 0.30 | 0.43 | 2.90 x 10^-06^ | 0.58 (0.46-0.73) |

Chr = chromosome, MAF = minor allele frequency, RR = relative risk, CI = confidence Interval

^1^ the underlined allele represents the GC risk allele

**2b)** Case control sample with Lithuanian origin (159 GC cases, 180 controls)

| **SNP** | **Chr** | **Allele^1^** | **MAF (%) cases** | **MAF (%) controls** | **p-value** | **RR (95% CI)** |
| --- | --- | --- | --- | --- | --- | --- |
| rs7716982 | 5 | G/T | 0.33 | 0.33 | 8.22 x 10^-01^ | 1.04 (0.75-1.43) |
| rs6893430 | 5 | T/C | 0.15 | 0.18 | 2.12 x 10^-01^ | 0.77 (0.51-1.16) |
| rs6861121 | 5 | G/A | 0.10 | 0.08 | 5.70 x 10^-01^ | 1.18 (0.69-2.01) |
| rs7726237 | 5 | G/A | 0.35 | 0.29 | 1.19 x 10^-01^ | 1.30 (0.94-1.80) |
| rs10737963 | 5 | C/A | 0.24 | 0.22 | 4.83 x 10^-01^ | 1.14 (0.79-1.63) |
| rs12523329 | 5 | C/T | 0.27 | 0.27 | 9.58 x 10^-01^ | 0.99 (0.71-1.39) |
| rs1002424 | 5 | G/A | 0.20 | 0.28 | 1.48 x 10^-02^ | 0.64 (0.44-0.91) |
| rs257009 | 5 | C/T | 0.31 | 0.27 | 2.88 x 10^-01^ | 1.21 (0.86-1.69) |
| rs10053664 | 5 | C/T | 0.50 | 0.45 | 2.29 x 10^-01^ | 1.21 (0.89-1.63) |
| rs13361707 | 5 | T/C | 0.20 | 0.28 | 1.51 x 10^-02^ | 0.64 (0.45-0.91) |
| rs3805486 | 5 | G/A | 0.11 | 0.13 | 5.50 x 10^-01^ | 0.87 (0.55-1.39) |
| rs462366 | 5 | T/C | 0.25 | 0.24 | 7.03 x 10^-01^ | 1.07 (0.75-1.52) |
| rs6876367 | 5 | T/C | 0.18 | 0.26 | 1.76 x 10^-02^ | 0.63 (0.44-0.91) |
| rs2291782 | 5 | A/G | 0.34 | 0.34 | 9.83 x 10^-01^ | 1.00 (0.73-1.38) |
| rs2976400 | 8 | A/G | 0.35 | 0.28 | 3.16 x 10^-02^ | 1.43 (1.03-1.99) |
| rs2976392 | 8 | G/A | 0.38 | 0.45 | 7.02 x 10^-02^ | 0.76 (0.56-1.03) |
| rs2976397 | 8 | G/T | 0.38 | 0.46 | 4.44 x 10^-02^ | 0.74 (0.54-1.00) |
| rs12155758 | 8 | A/G | 0.42 | 0.35 | 6.44 x 10^-02^ | 1.33 (0.98-1.82) |
| rs1435453 | 8 | T/C | 0.35 | 0.40 | 2.12 x 10^-01^ | 0.83 (0.60-1.13) |

Chr = chromosome, MAF = minor allele frequency, RR = relative risk, CI = confidence Interval

^1^ the underlined allele represents the GC risk allele

**2c)** Case control sample with Spanish origin (529 GC cases, 569 controls)

| **SNP** | **Chr** | **Allele^1^** | **MAF (%) cases** | **MAF (%) controls** | **p-value** | **RR (95% CI)** |
| --- | --- | --- | --- | --- | --- | --- |
| rs7716982 | 5 | G/T | 0.34 | 0.32 | 2.17 x 10^-01^ | 1.12 (0.94-1.34) |
| rs6893430 | 5 | T/C | 0.21 | 0.22 | 5.16 x 10^-01^ | 0.94 (0.76-1.15) |
| rs6861121 | 5 | G/A | 0.15 | 0.16 | 5.39 x 10^-01^ | 0.93 (0.74-1.17) |
| rs7726237 | 5 | G/A | 0.33 | 0.30 | 2.65 x 10^-01^ | 1.11 (0.93-1.33) |
| rs10737963 | 5 | C/A | 0.27 | 0.23 | 6.70 x 10^-02^ | 1.21 (1.00-1.47) |
| rs12523329 | 5 | C/T | 0.26 | 0.25 | 7.29 x 10^-01^ | 1.04 (0.86-1.26) |
| rs1002424 | 5 | G/A | 0.28 | 0.30 | 2.44 x 10^-01^ | 0.89 (0.74-1.08) |
| rs257009 | 5 | C/T | 0.31 | 0.28 | 2.96 x 10^-01^ | 1.11 (0.92-1.33) |
| rs10053664 | 5 | C/T | 0.42 | 0.41 | 7.27 x 10^-01^ | 1.03 (0.87-1.22) |
| rs13361707 | 5 | T/C | 0.28 | 0.30 | 2.13 x 10^-01^ | 0.89 (0.74-1.07) |
| rs3805486 | 5 | G/A | 0.15 | 0.17 | 2.87 x 10^-01^ | 0.88 (0.70-1.11) |
| rs462366 | 5 | T/C | 0.26 | 0.24 | 2.20 x 10^-01^ | 1.13 (0.93-1.38) |
| rs6876367 | 5 | T/C | 0.29 | 0.31 | 5.20 x 10-^01^ | 0.94 (0.78-1.13) |
| rs2291782 | 5 | A/G | 0.29 | 0.29 | 7.75 x 10^-01^ | 1.03 (0.85-1.24) |
| rs2976400 | 8 | A/G | 0.35 | 0.33 | 3.23 x 10^-01^ | 1.09 (0.92-1.31) |
| rs2976392 | 8 | A/G | 0.49 | 0.46 | 1.66 x 10^-01^ | 1.13 (0.95-1.33) |
| rs2976397 | 8 | T/G | 0.47 | 0.44 | 1.03 x 10^-01^ | 1.15 (0.97-1.36) |
| rs12155758 | 8 | A/G | 0.25 | 0.26 | 5.71 x 10^-01^ | 0.95 (0.78-1.15) |
| rs1435453 | 8 | T/C | 0.44 | 0.45 | 5.13 x 10^-01^ | 0.95 (0.80-1.12) |

Chr = chromosome, MAF = minor allele frequency, RR = relative risk, CI = confidence Interval

^1^ the underlined allele represents the GC risk allele

**2d)** Case control sample with German origin (942 GC cases, 925 controls)

| **SNP** | **Chr** | **Allele^1^** | **MAF (%) cases** | **MAF (%) controls** | **p-value** | **RR (95% CI)** |
| --- | --- | --- | --- | --- | --- | --- |
| rs7716982 | 5 | G/T | 0.35 | 0.34 | 5.84 x 10^-01^ | 1.04 (0.91-1.19) |
| rs6893430 | 5 | T/C | 0.18 | 0.20 | 9.89 x 10^-02^ | 0.87 (0.74-1.03) |
| rs6861121 | 5 | G/A | 0.11 | 0.12 | 8.72 x 10^-02^ | 0.84 (0.68-1.03) |
| rs7726237 | 5 | G/A | 0.33 | 0.32 | 2.96 x 10^-01^ | 1.08 (0.94-1.23) |
| rs10737963 | 5 | C/A | 0.26 | 0.25 | 8.57 x 10^-01^ | 1.01 (0.88-1.18) |
| rs12523329 | 5 | C/T | 0.27 | 0.25 | 1.58 x 10^-01^ | 1.11 (0.96-1.29) |
| rs1002424 | 5 | G/A | 0.25 | 0.27 | 1.53 x 10^-01^ | 0.90 (0.78-1.04) |
| rs257009 | 5 | C/T | 0.31 | 0.30 | 5.01 x 10^-01^ | 1.05 (0.91-1.21) |
| rs10053664 | 5 | C/T | 0.44 | 0.43 | 5.48 x 10^-01^ | 1.04 (0.91-1.18) |
| rs13361707 | 5 | T/C | 0.25 | 0.27 | 1.39 x 10^-01^ | 0.90 (0.78-1.04) |
| rs3805486 | 5 | G/A | 0.12 | 0.13 | 1.15 x 10^-01^ | 0.86 (0.70-1.04) |
| rs462366 | 5 | T/C | 0.27 | 0.26 | 9.00 x 10^-01^ | 1.01 (0.87-1.17) |
| rs6876367 | 5 | T/C | 0.26 | 0.27 | 3.45 x 10^-01^ | 0.93 (0.81-1.08) |
| rs2291782 | 5 | A/G | 0.30 | 0.28 | 3.30 x 10^-01^ | 1.07 (0.93-1.24) |
| rs2976400 | 8 | A/G | 0.32 | 0.32 | 8.91 x 10^-01^ | 0.99 (0.86-1.14) |
| rs2976392 | 8 | A/G | 0.49 | 0.46 | 4.00 x 10^-04^ | 1.26 (1.11-1.43) |
| rs2976397 | 8 | T/G | 0.49 | 0.44 | 4.73 x 10^-04^ | 1.26 (1.10-1.43) |
| rs12155758 | 8 | A/G | 0.32 | 0.27 | 2.98 x 10^-04^ | 1.30 (1.13-1.49) |
| rs1435453 | 8 | T/C | 0.42 | 0.47 | 3.40 x 10^-03^ | 0.83 (0.73-0.94) |

Chr = chromosome, MAF = minor allele frequency, RR = relative risk, CI = confidence Interval

^1^ the underlined allele represents the GC risk allele

**Supplementary Table 3:** Case control comparison (1,926 GC cases, 2,012 controls) of genotyped SNPs at chromosomal regions 5p13 and 8q24.

**3a)** Case control sample with Latvian origin (296 GC cases, 338 controls)

| **SNP** | **Chr** | **Allele^1^** | **MAF (%) cases** | **MAF (%) controls** | **p-value** | **RR (95% CI)** |
| --- | --- | --- | --- | --- | --- | --- |
| rs6872282 | 5 | T/C | 0.22 | 0.26 | 7.32 x 10^-02^ | 1.27 (0.98-1.64) |
| rs2585176 | 8 | A/T | 0.37 | 0.50 | 1.45 x 10^-07^ | 1.93 (1.50-2.48) |

Chr = chromosome, MAF = minor allele frequency, RR = relative risk, CI = confidence Interval

^1^ the underlined allele represents the GC risk allele

**3b)** Case control sample with Lithuanian origin (159 GC cases, 180 controls)

| **SNP** | **Chr** | **Allele^1^** | **MAF (%) cases** | **MAF (%) controls** | **p-value** | **RR (95% CI)** |
| --- | --- | --- | --- | --- | --- | --- |
| rs6872282 | 5 | T/C | 0.16 | 0.27 | 1.30 x 10^-03^ | 1.83 (1.25-2.67) |
| rs2585176 | 8 | A/T | 0.40 | 0.48 | 3.27 x 10^-02^ | 1.42 (1.03-1.97) |

Chr = chromosome, MAF = minor allele frequency, RR = relative risk, CI = confidence Interval

^1^ the underlined allele represents the GC risk allele

**3c)** Case control sample with Spanish origin (529 GC cases, 569 controls)

| **SNP** | **Chr** | **Allele^1^** | **MAF (%) cases** | **MAF (%) controls** | **p-value** | **RR (95% CI)** |
| --- | --- | --- | --- | --- | --- | --- |
| rs6872282 | 5 | T/C | 0.26 | 0.28 | 2.12 x 10^-01^ | 1.13 (0.93-1.36) |
| rs2585176 | 8 | T/A | 0.46 | 0.43 | 1.31 x 10^-01^ | 1.14 (0.96-1.36) |

Chr = chromosome, MAF = minor allele frequency, RR = relative risk, CI = confidence Interval

^1^ the underlined allele represents the GC risk allele

**3d)** Case control sample with German origin (942 GC cases, 925 controls)

| **SNP** | **Chr** | **Allele^1^** | **MAF (%) cases** | **MAF (%) controls** | **p-value** | **RR (95% CI)** |
| --- | --- | --- | --- | --- | --- | --- |
| rs6872282 | 5 | T/C | 0.23 | 0.25 | 3.98 x 10^-02^ | 1.18 (1.01-1.37) |
| rs2585176 | 8 | T/A | 0.49 | 0.42 | 7.13 x10^-05^ | 1.32 (1.15-1.51) |

Chr = chromosome, MAF = minor allele frequency, RR = relative risk, CI = confidence Interval

^1^ the underlined allele represents the GC risk allele

**Supplementary Table 4:** Genotype-phenotype (GxP) analysis of rs6872282 at chromosome 5p13 using GC tumor localization (cardia, non-cardia) and Lauren type (diffuse, intestinal) as strata.

| **Top hit of the imputation** | **all**  **RR (95 % CI)** | **non-cardia**  **RR (95 % CI)** | **cardia**  **RR (95 % CI)** | **diffuse**  **RR (95 % CI)** | **intestinal**  **RR (95 % CI)** |
| --- | --- | --- | --- | --- | --- |
| rs6872282 | 1.22 (1.09 - 1.35) | 1.21 (1.07 - 1.37) | 1.09 (0.89 - 1.32) | 1.25 (1.07 - 1.46) | 1.11 (0.96 - 1.29) |

RR = relative risk, CI = confidence interval

**Supplementary Table 5:** Genotype-phenotype (GxP) analysis of rs258176 at chromosome 8q24 using GC tumor localization (cardia, non-cardia) and Lauren type (diffuse, intestinal) as strata.

| **Top hit of the imputation** | **all**  **RR (95 % CI)** | **non-cardia**  **RR (95 % CI)** | **cardia**  **RR (95 % CI)** | **diffuse**  **RR (95 % CI)** | **intestinal**  **RR (95 % CI)** |
| --- | --- | --- | --- | --- | --- |
| rs2585176 | 1.34 (1.22 - 1.47) | 1.31 (1.17 - 1.46) | 1.14 (0.96 - 1.36) | 1.31 (1.14 - 1.51) | 1.27 (1.11 - 1.44) |

RR = relative risk, CI = confidence interval

**Supplementary Table 6:** Top associated SNPs at chromosome 5p13 and corresponding traits as well as their LD (r^2^) to rs6872282, the most associated GC SNP in the present study

| **SNP** | **Chr** | **Position (hg19)** | **r² to rs6872282** | **Associated Trait/Disease** | **Study** |
| --- | --- | --- | --- | --- | --- |
| rs4613763 | 5 | 40392727 | 0.034 | Crohn's Disease | Barrett et al. Nature Genetics 2008 40: 955-62 |
| rs17234657 | 5 | 40401508 | 0.034 | Crohn's Disease | WTCCC. Nature 2007 447: 661-78 |
| rs11742570 | 5 | 40410583 | 0.146 | Crohn's Disease | Jostins et al. Nature 2012 491: 119-24 |
| rs6451493 | 5 | 40410934 | 0.146 | Ulcerative Colitis | Anderson et al. Nature Genetics 2011 43: 246-52 |
| rs6896969 | 5 | 40424425 | 0.215 | Multiple Sclerosis | De Jager et al. Nature Genetics 2009 41: 776-82 |
| rs1373692 | 5 | 40431182 | 0.192 | eQTL in lymphoblastoid cell lines | Libioulle et al. PLoS Genetics 2007 3: e58 |
| rs9292777 | 5 | 40437947 | 0.207 | Multiple Sclerosis | Matesanz et al. PloS One 2012 7: e36140 |
| rs10440635 | 5 | 40490789 | 0.197 | Ankylosing Spondylitis | Evans et al. Nature Genetics 2011 43: 761-7 |
|  |  |  |  |  | Julia et al. Gut 2013 62: 1440-5 |
|  |  |  |  |  | Franke et al. Nature Genetics 2010 42: 1118-25 |
|  |  |  |  |  | Weersma et al. The American Journal of Gastroenterology 2009 104: 630-8 |
|  |  |  |  | Ulcerative Colitis | Latiano et al. PloS One 2011 6: e22688 |
|  |  |  |  |  | McGovern et al. Nature Genetics 2010 42: 332-7 |
|  |  |  |  | Multiple Sclerosis | Sawcer et al. Nature 2011 476: 214-9 |

Chr = chromosome
